# Quality assessment of Chinese liquor with different ages and prediction analysis based on gas chromatography and electronic nose

**DOI:** 10.1038/s41598-017-06958-7

**Published:** 2017-07-26

**Authors:** M. L. Xu, S. M. Zhu, Y. Yu

**Affiliations:** 1 College of Biosystems Engineering and Food Science, Zhejiang University, Hangzhou, 310058 China; 2 Key laboratory of Equipment and Information in Environment Controlled Agriculture, Ministry of Agriculture, Hangzhou, 310058 China

## Abstract

The economic value of Chinese liquor is closely related with its age. Results from gas chromatograph (GC) analysis indicated that 8 dominant compounds were decreased with the increase of liquor age (0 to 5 years) while ethyl lactate was found to be the most stable dominant compound as no significant change was observed in it during the aging process. Liquor groups with different ages were well-discriminated by principal component analysis (PCA) based on electronic nose signals. High-accurate identification of liquor ages was realized using linear discriminant analysis (LDA) with the accuracy of 98.3% of the total 120 samples from six age groups. Partial least squares regression (PLSR) exhibited satisfying ability for liquor age prediction (R^2^: 0.9732 in calibration set and 0.9101 in validation set). The feasibility of volatile compounds prediction using PLSR combined with electronic nose was also verified by this research. However, the accuracies of PLSR models can be further promoted in future researches, perhaps by using more suitable sensors or modeling approaches.

## Introduction

Chinese liquor is one kind of traditional alcoholic beverages with a long history of over 6000 years^1^. The sales volume of Chinese liquor reached 82 billion dollars in 2015 which played an important role in the national economic development. Though small differences may exist in the manufacturing techniques of various liquors depending on the producing area and flavor type, three essential processes are generally involved: fermentation, distillation and aging^2^. Newly distilled liquors (young liquors) are often subjected to several months to years of aging to remove the undesired pungent flavor. During this aging process, a series of complex physicochemical reactions such as oxidation, esterification and hydrolysis can take place along with the possible permeation of small and polar molecules through the ceramic jar^3, 4^. Since the economic value of Chinese liquor is highly associated with its age, some dishonest producers counterfeit their younger products as several years aged liquors to get a higher selling price. Thus, the discrimination of liquor age and related predictive analysis are urgently needed to safeguard the market order and to protect consumer rights.

The current strategies which have been used to identify liquor age can be divided into two categories: sensorial evaluation and instrumental analysis. Sensorial evaluations are usually conducted by well-trained analysts to grade Chinese liquor according to characteristics of color, aroma, taste and overall quality^5, 6^. It is obvious that the evaluation result is lack of objectivity and reproducibility to some extent, since it can be easily affected by external conditions of the environment and internal physical and mental status of the analysts^7^. As for the instrumental analysis, gas chromatography^8^ or a combination of gas chromatography and mass spectrometry^9^, high performance liquid chromatography^10^, atomic absorption spectroscopy^11^ and spectrograph technique^12^ have been widely applied to analyze flavor characteristics of Chinese liquor based on their volatile compounds. However, in order to acquire accurate results, sophisticated pretreatment, long experimental period and skilled operating technician are usually needed, which causes much problem to research work. Above all, it’s of great importance to find out an ideal method to characterize flavor features of Chinese liquor precisely and conveniently in relation to the determination of liquor age.

During the last decades, more and more attention has been focused on the development of electronic nose, a non-destructive and rapid method used for detecting aroma characteristics of food matrix. The core component of electronic nose system is the sensor array; each sensor generates specific response to corresponding aroma substance exists in the headspace of food samples, with the purpose of simulating human nose. Then a data-collection unit is usually used to acquire response values of the sensor array, after that, discrimination and prediction analysis can be realized by using multivariate statistical techniques. Apart from low cost and timesaving features, electronic nose has also been proved to perform well in volatile compounds analysis of various foods, including fruit juices^13, 14^, alcoholic beverages^15, 16^ and green tea^17^.

Chinese liquors with different ages were used in this study, and nine dominant volatile compounds were selected and researched. The main objectives of this study were: (1) to investigate the possibility of using electronic nose combined with principal component analysis (PCA) and linear discriminant analysis (LDA) to discriminate Chinese liquor with different ages; (2) to realize prediction analysis of liquor age and the contents of main volatile compounds in Chinese liquor based on response values and partial least squares regression (PLSR).

## Results

### Quantification of volatile compounds

Although hundreds of volatile compounds have been found in Chinese liquor by previous researchers, only the dominant compounds were selected in this work, since the main objective of this research was to explore the possibility of predicting volatile compounds contents using electronic nose signals. As shown in Table 1, within the nine dominant compounds, 2 aldehydes, 4 alcohols, 2 esters and 1 acid were included, and changes of them during aging were also characterized.

**Table 1:** Concentration of major volatile compounds in different aged Chinese liquors.

Number	Compounds	Young	One year	Two years	Three years	Four years	Five years
1	Acetaldehyde	237.81 ± 11.87a	152.99 ± 12.67b	148.14 ± 6.34b	144.73 ± 13.72b	153.67 ± 5.76b	118.96 ± 4.53c
2	Methanol	79.79 ± 3.46a	46.75 ± 2.06b	41.09 ± 1.66b	44.62 ± 3.97b	44.68 ± 2.35b	39.86 ± 1.11b
3	Ethyl acetate	1741.48 ± 87.52a	694.23 ± 36.17b	677.85 ± 44.60b	692.20 ± 164.33b	627.80 ± 32.33b	446.29 ± 20.55c
4	Acetal	519.72 ± 30.60a	275.31 ± 22.76b	260.37 ± 11.09b	267.91 ± 23.17b	255.42 ± 18.14b	183.57 ± 6.46c
5	1-Propanol	338.84 ± 16.54a	261.24 ± 20.35b	247.04 ± 7.04b	267.68 ± 23.10b	270.81 ± 13.65b	341.57 ± 10.96a
6	Isobutanol	637.67 ± 39.72a	593.72 ± 48.15a	573.37 ± 29.01a	551.78 ± 39.16a	549.03 ± 35.42a	390.93 ± 22.71b
7	Isoamylol	800.53 ± 41.79a	793.02 ± 38.19a	766.17 ± 19.15a	740.56 ± 52.14a	744.52 ± 44.75a	588.39 ± 18.53b
8	Ethyl lactate	446.38 ± 30.40a	465.10 ± 31.16a	445.73 ± 27.98a	450.21 ± 19.36a	454.67 ± 31.46a	443.85 ± 23.94a
9	Acetic acid	1194.00 ± 91.82a	701.73 ± 40.19b	688.29 ± 37.69b	673.71 ± 44.96b	676.13 ± 38.74b	570.52 ± 30.31c

All values are expressed as means (mg/L) ± standard deviation (SD).

Different letters indicate significant differences (p < 0.05).

Aldehydes are generally formed by the oxidization of alcohols present in food and are usually described as having the odor of overripe apples^18, 19^. As presented in Table 1, concentrations of acetaldehyde and acetal were decreased rapidly during the first year of aging with the rate of 35% and 47% (P < 0.05), respectively, and no significant change was found in the following three years. However, a slight decrease (P < 0.05) was both observed in the fifth year, which could conduce to human health since high concentration of acetal can cause damage to liver. Alcohols are mainly produced by deamination of amino acids under anaerobic conditions and/or decarboxylation reactions of sugars under aerobic conditions during the fermentation process^20^. As can be seen, methanol and 1-propanol decreased significantly in the first year and maintained at the same level (P > 0.05) in the following years, while the reduction of isoamylol and isobutanol contents (P < 0.05) were only observed in the fifth year. It should be noted that the control of methanol level is of great importance in the manufacturing process of Chinese liquor due to its toxicity, though the content is generally much lower than other abundant alcohols. As for esters, the content of ethyl acetate was reduced to 40% within the first two years and then kept at the same level (P > 0.05) in the next three years, however, further reduction was found in the fifth year with the rate of 30%. As an exception, ethyl lactate seems to be the most stable compound since no significant change was observed throughout the researched aging process from the first year to fifth year. This relative stable content of ethyl lactate can conduce to the mellow sensorial properties of Chinese liquor. The concentration of acetic acid was 1194 mg/L, which contributed more than 90% of the acidity of Chinese liquor. In the first year, a 41% decline from 1194 to 701 mg/L was found, after that, no significant change was observed until the fifth year. Acids in Chinese liquor can have impact on sensory characteristics, contribute to color stability and increase antioxidant power of the products^21^.

Above all, eight compounds were found to decrease during the aging process except for ethyl acetate. Besides the complicated physicochemical reactions, it can be inferred that volatilization and the possible permeation might also have played an indispensable role during this process.

### Electronic nose analysis and selection of response values

**Figure 1:**
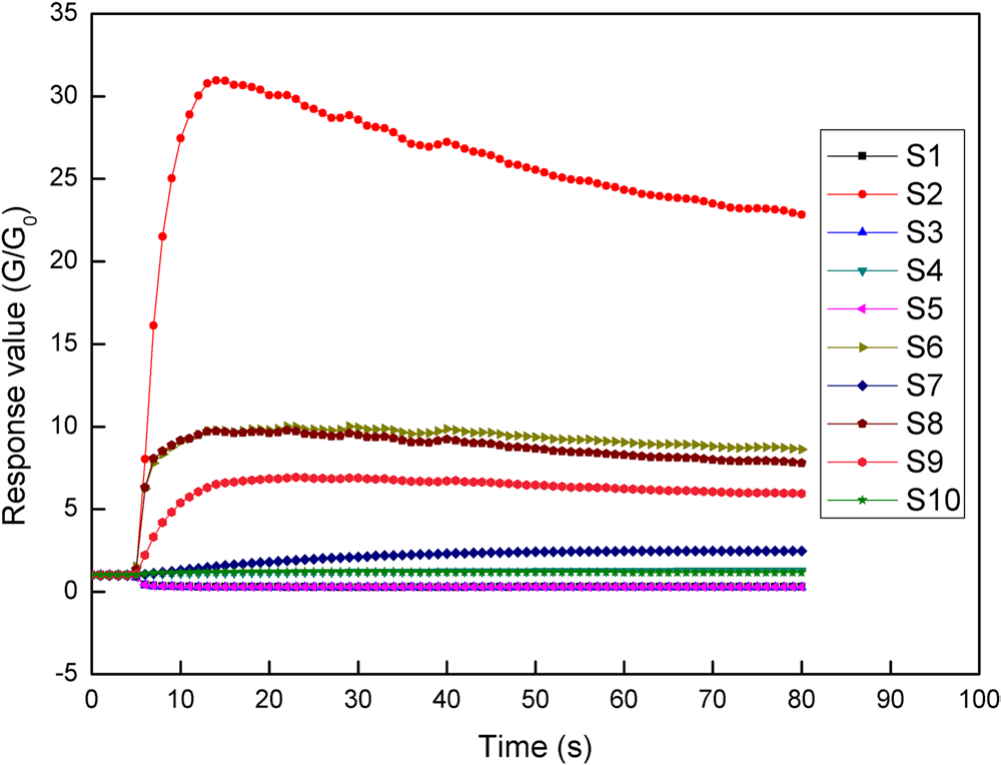
Typical responding curves of liquor samples obtained from electronic nose.

The response value of sensors was calculated as G/G_0_, where G_0_ is defined as the electric conductivity of nitrogen (cleaning gas), and G is defined as the electric conductivity of tested samples. Typical response curves of the sensor array during the detection period of liquor samples are given in Fig. 1. The initial response values of ten sensors were all around 1, which means that the sensor array was well-calibrated before the detection. This process went on for about five seconds, and then response values of almost all sensors diverged from 1 and gradually tended to be stable after 40 s except for S2. Considering the continuous decrease of S2, the response values of all sensors at the 70th second were chosen as the original data. As can be seen from Table 2, low RSD values and significant differences (p < 0.001) obtained for all sensors among six liquor groups with different ages indicated that response values of the sensor array were stable and credible when exposed to liquor samples, and all the 10 sensors were closely related with liquor ages. Thus, response values of all sensors (S1 to S10) were used as the data matrix for the following discrimination and prediction analysis.

**Table 2:** Results of RSD test and One Way ANOVA of response values.

Sensors	Young (%)	One (%)	Two (%)	Three (%)	Four (%)	Five (%)	F value	P value
S1	2.21	1.96	2.76	2.04	2.29	2.08	73.088	<0.001
S2	4.19	3.07	4.60	3.50	2.44	3.00	39.975	<0.001
S3	2.47	1.93	2.51	2.10	2.32	2.16	22.989	<0.001
S4	1.51	1.91	1.84	1.86	2.25	1.03	104.818	<0.001
S5	2.40	1.84	2.39	2.02	2.14	1.91	20.253	<0.001
S6	5.47	3.80	6.19	4.37	4.28	3.86	34.211	<0.001
S7	2.70	1.97	3.77	2.80	2.81	2.29	8.283	<0.001
S8	7.12	5.36	7.66	5.82	4.23	3.52	59.781	<0.001
S9	3.42	3.01	3.58	2.71	2.69	2.52	25.731	<0.001
S10	1.08	0.80	1.30	0.97	0.94	0.47	46.629	<0.001

Each RSD value was obtained from 20 samples of each group. One Way ANOVA of each sensor was performed with 120 samples (20 replicates multiply by 6 groups).

### Classification based on principal components analysis (PCA)

**Figure 2:**
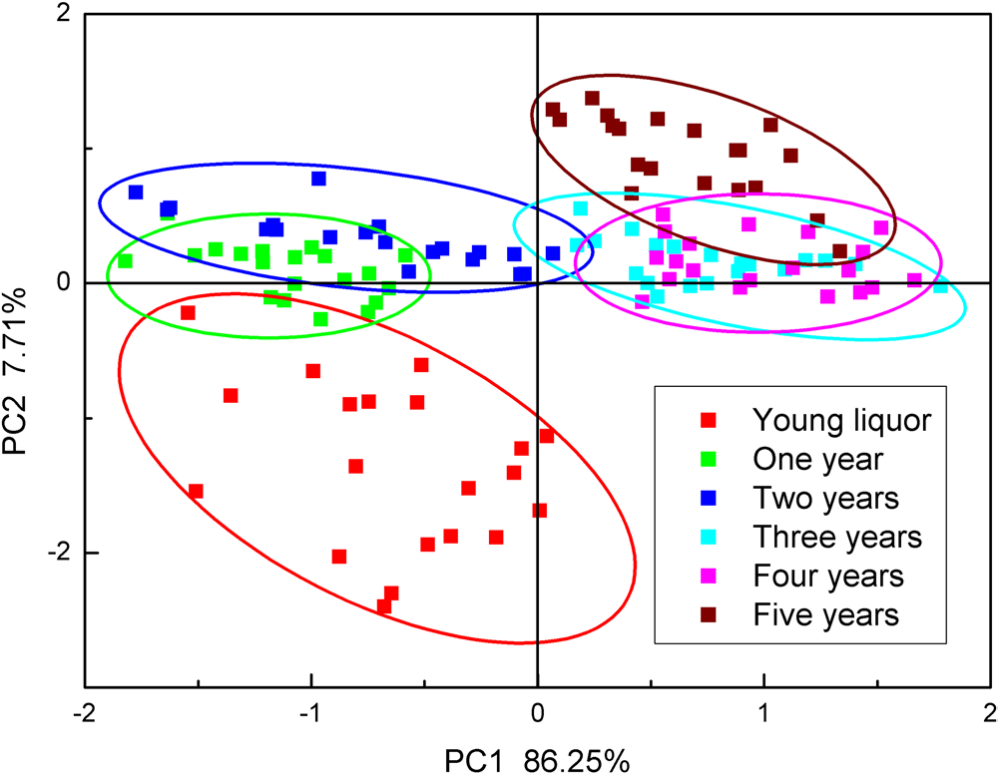
PCA results of liquor samples with different age based on response values.

As shown in the scores plot (Fig. 2), the first two principal components (PC1 & PC2) were taken as coordinate axes, which explained the total variance of 90.36% (82.65% for PC1 and 7.71% for PC2). It showed that liquor samples clustered closely together within group and separated clearly from each other between groups. Specifically, young liquors without aging were grouped on the negative side of PC2 while the other five aged groups were clustered on the positive side of PC2. It seemed that PC2 played a dominant role in discriminating liquors with and without aging process rather than PC1. As for the five aged groups, one-year-aged and two-year-aged groups were located on the negative side of PC1 while the other three aged groups were situated on the positive side.

Young liquors clustered individually as one category suggested the existence of big overall differences between young and aged liquors. The overlapped region between one-year and two-year aged liquors were resulted from the similar volatile composition in these two groups. During the second year of aging, only small changes were observed in the main volatile compounds, and as a consequence, similar response values were obtained in these two liquor groups, thus it can be hard to completely discriminate one-year-aged liquor from two-year-aged one based on the response values of electronic nose. Similar phenomenon was also observed between three-year and four-year aged samples, which was caused by the same reason as previously stated. Only three samples from five-year-aged liquors were distributed within the region of four-year-aged group indicating a satisfying separation, and this was mainly due to the further significant changes that took place in the fifth year of aging. These findings can be verified by the results of gas chromatography analysis. As stated previously, the concentrations of volatile compounds in two-year and three-year aged liquors were similar to each other, but quite unexpectedly, these two groups were located on the different side of PC1, namely, two-year for the negative side while three-year for the positive side, which elucidated the existence of big overall gaps between them. This might have been caused by some undetected substance which strongly affected the sensor responses of electronic nose; however, further research should be carried out to investigate what exactly happened during the third year of aging.

### Identification based on linear discriminant analysis (LDA)

As discussed before, classification based on PCA presented some partial overlaps between groups, thus, LDA was carried out to realize a better discrimination as it has been proved to perform well in the identification of Chinese liquor with different aroma characteristics^22^ and green tea produced in various areas^23^. Mahalanobis Distance Stepwise method was used and the F value of 3.84 was set as the joining condition of all sensors, therefore, only sensors with F values higher than 3.84 could be chosen as subsequent discriminating factors. As a result, S5, S7 and S8 were removed since the F values of them were lower than 3.84 (1.93, 3.10 and 1.56, respectively).

**Figure 3:**
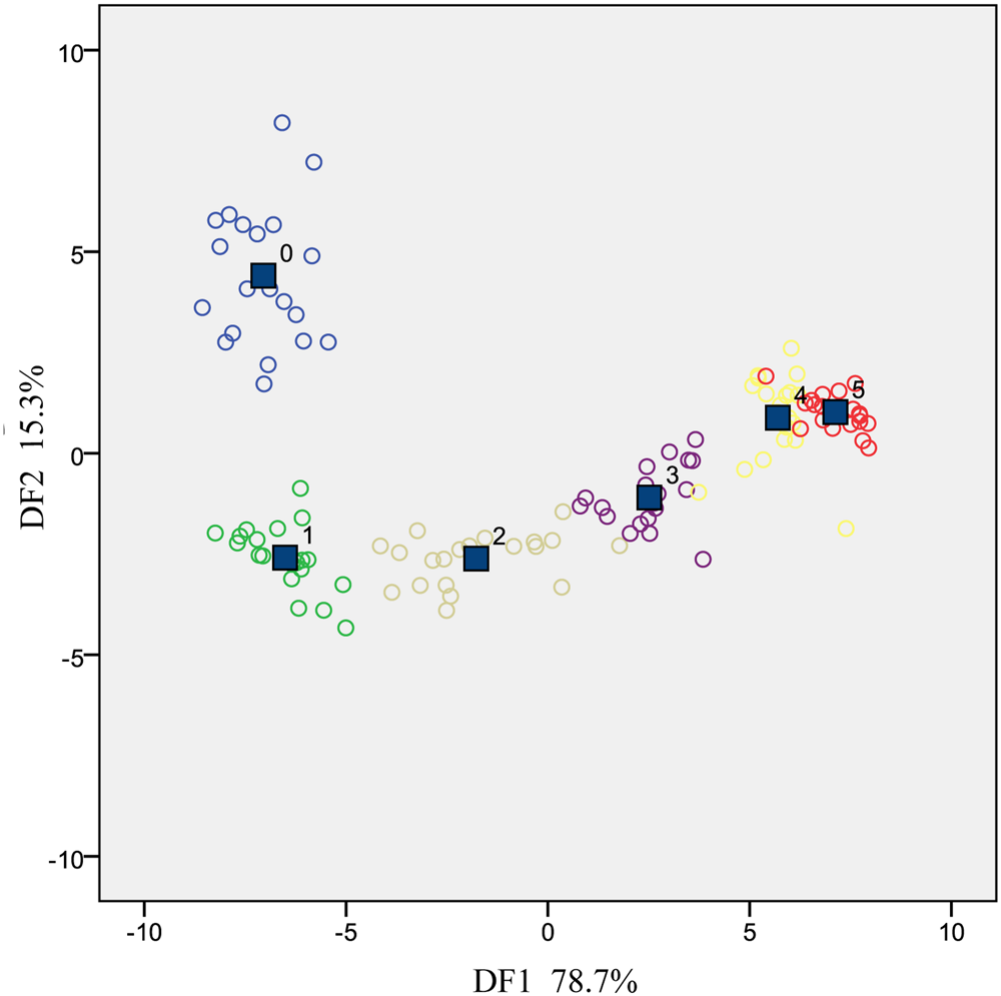
LDA results of liquor samples with different ages based on response values.

Figure 3 displays the distribution of six groups based on discriminant functions. The first discriminant function (DF1) and the second discriminant function (DF2) were selected as coordinate axes, which explained 94.0% of the total variance (78.7% for DF1 and 15.3% for DF2). The classification results of 120 liquor samples are presented in Table 3. As can be seen, only two liquor samples were wrongly identified, one from two-year-aged group was recognized as three-year-aged liquor, the other one from four-year-aged group was graded as five-year-aged liquor. This also could be observed from Fig. 3, the unexpected distributions of these two samples might have been caused by the fluctuations of one or several sensors. Beyond that, the rest of 118 liquor samples were all correctly classified, and reached a total accuracy of 98.3%. These results confirmed the feasibility of using LDA based on electronic nose to realize a highly accurate identification of Chinese liquor with different ages. In addition, the better identification results achieved by LDA in comparison with PCA indicated the importance of sensor optimization, since all sensors were used in PCA while less relevant sensors (S5, S7 and S8) were excluded in LDA.

**Table 3:** Classification results of six age groups based on LDA.

Actual age	Predicted age						Accuracy
	0	1	2	3	4	5	
0	20						100%
1		20					100%
2			19	1			95%
3				20			100%
4					19	1	95%
5						20	100%

The total accuracy of the 120 samples is 98.3%.

### Prediction of liquor age

The prediction analysis of liquor age was performed by PLSR. Responses values of Chinese liquor with different ages obtained from 10 sensors were used as the raw database; these values were directly applied to the prediction model as independent variables, while liquor ages were applied as dependent variables. 120 samples (20 from each group) were randomly divided into two sub-groups, one for calibration and the other one for validation. The calibration set consisted of 90 samples (15 from each group) and the validation set contained 30 samples (5 from each group).

To isolate a few potential sensor values that provide a good prediction work, a PLSR model was firstly fitted using the 90 samples from validation set, as a result, ten factors were extracted. After that, leave-one-out technique was carried out for the cross validation in order to choose the suitable number of factors. During that process, 90 samples from the calibration set was randomly divided into several sub-groups, all the sub-groups were used to fit a PLSR model except one, then the capability of the fitted model to predict response values for the omitted group was evaluated. The above process was then repeated for each sub-group, and the overall capability of a given form of the model could be measured by comparing the predicted residual sum of squares (PRESS) based on the residuals generated by this process. The minimum PRESS of 0.1980 was acquired with six extracted factors; however, PRESS values obtained with three, four and five factors (0.2536, 0.2039 and 0.2092) were close to the one of six factors, which suggested that six factors might not be the best choice. To verify this conjecture, a statistical model comparison suggested by van der Voet^24^ was performed to test whether the differences were significant. The p value of 0.544 in comparing the cross-validated residuals from models with six and four factors indicated that the difference between the two models was insignificant, while the one with six and three was significant. Therefore, four factors were finally confirmed as the smallest number of factors with the minimum PRESS. The selected four factors accounted for 95.6% of the total variances for the model (85.9%, 5.8%, 2.4% and 1.4%, respectively) and 96.8% for the dependent variables (58.3%, 32.5%, 4.6% and 1.3%, respectively). We hereto acquired a four-factor PLSR model for predicting liquor ages based on response values obtained from electronic nose.

**Figure 4:**
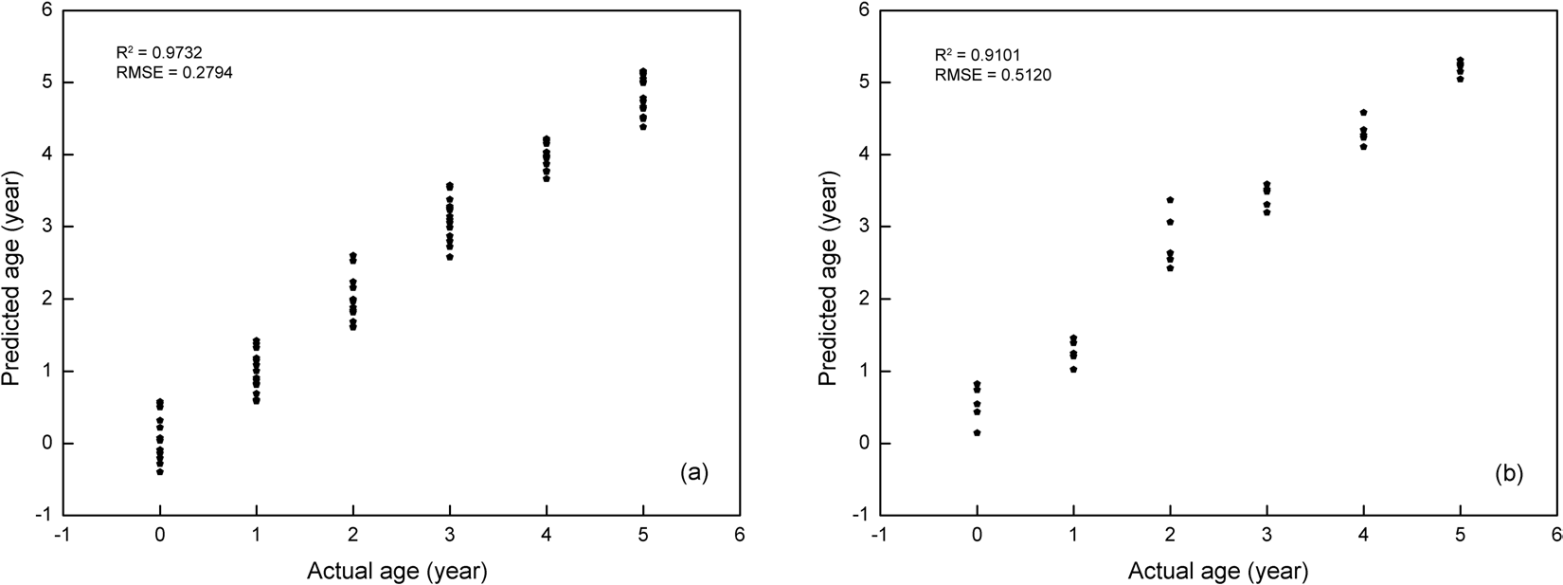
PLSR results for the prediction of liquor age. (**a**): calibration set, (**b**): validation set.

The calibration and validation results are displayed in Fig. 4(a) and (b) with the form of scatter diagram. The fitting degree of PLSR model (R^2^) was calculated based on the actual liquor age and the PLSR predicted liquor age. What should be emphasized was that the regression coefficient (R^2^) of the liner regression model between the actual and predicted liquor ages was not the same one as PLSR model; so it can only be used to express the fitting degree of the liner regression model, not the PLSR model, and that was the reason why scatter diagram was used rather than liner fitting diagram. As for the calibration set, the regression coefficient R^2^ between the actual liquor age and predicted liquor age was 0.9732, which exhibited a good correlation. The root mean square error (RMSE) of the calibration set was 0.2794. In order to have a further understanding of the calibration results, RMSE values of different groups (15 samples for each group) were separately calculated and compared. RMSE values for young liquor, one-year-aged, two-year-aged, three-year-aged, four-year-aged and five-year-aged liquors were 0.3076, 0.2793, 0.2945, 0.3013, 0.1736 and 0.2970, respectively. All values were around the average RSME (0.2794) except for four-year-aged liquors, it could be concluded that the most accurate prediction was achieved with four-year-aged liquor in the calibration set, since the RMSE values of other five groups were quite higher when compared with four-year-aged one. As for the validation set, only 30 liquor samples were used. The regression coefficient R^2^ between the actual liquor age and predicted liquor age in validation set was 0.9101, which logically explained a satisfying prediction ability of the liquor age. The RSME of validation set was calculated as 0.5120, which was much higher than calibration set. That was reasonable because the correlation between liquor age and sensor response in calibration set was inevitably different with the one for validation set, so the calibration set generally fitted the model better than the validation set. Similar, the RSME values of each group (5 samples for each group) in validation set were also separately calculated and compared. RMSE values for young liquor, one-year-aged, two-year-aged, three-year-aged, four-year-aged and five-year-aged liquors were 0.5887, 0.3074, 0.8787, 0.4407, 0.3440 and 0.2180, respectively. Unlike the calibration set, the minimum RSME of validation set was obtained with five-year-aged liquor, in addition, RSME of one-year-aged and four-year-aged liquors were also much lower than the average value.

### Prediction of volatile compounds content

As previously discussed, of all the nine compounds researched, the content of ethyl lactate was kept in the range of 443 to 446 mg/L from the beginning to the end of the aging, and no significant difference was observed among six liquor groups with different ages. Based on that, the prediction of ethyl lactate content was meaningless at least during the first five years of aging, thus only eight compounds were studied in this part. For all the prediction modeling of eight compounds, 90 samples (15 samples for each group) were randomly selected as calibration set and the other 30 samples (5 samples for each group) were used as validation set. After the extraction of factors, cross-validation was also performed using leave-one-out technique to choose the optimized number of factors used for the subsequent modeling.

Calibration and validation results are listed in Table 4. The regression coefficient R^2^ between the actual and predicted values and the RMSE of each compounds were given. The total sum of the square was consisted of regression sum of square and residual sum of square, and the fitting degree (R^2^) of the PLSR model was calculated by the regression sum of square versus the total sum of square. Regression coefficients (R^2^) of eight PLSR models in calibration set were within the range from 0.8602 to 0.9379, which demonstrated a good correlation between the actual and predicted values. As for the validation set, the prediction ability of 1-propanol PLSR model was found to be poor with a quite low R^2^ of 0.5439, while R^2^s of other seven compounds were ranged from 0.7053 to 0.7614. As can be seen from Table 4, in the calibration set, R^2^s of 1-propanol, isobutanol and isoamylol were 0.8602, 0.8935 and 0.8775, respectively, while other five compounds were all around 0.9300. Furthermore, the relatively lower R^2^s were also obtained with these three compounds when compared with other five compounds, in validation set. It seemed that the response values of the studied sensors and modeling technique had disadvantages in predicting alcohols contents, except for methanol. Beyond that, the established method performed well in the prediction of acetaldehyde, ethyl acetate, acetal and acetic acid.

**Table 4:** Prediction results of volatile compounds based on PLSR.

Compounds	Calibration set		Validation set	
	R^2^	RMSE	R^2^	RMSE
Acetaldehyde	0.9327	9.60	0.7459	18.63
Methanol	0.9379	3.43	0.7603	6.74
Ethyl acetate	0.9304	111.79	0.7542	210.13
Acetal	0.9343	27.04	0.7614	51.54
1-Propanol	0.8602	14.12	0.5439	25.50
Isobutanol	0.8935	25.08	0.7106	41.35
Isoamylol	0.8775	24.81	0.7053	38.49
Acetic acid	0.9354	55.51	0.7574	99.89

R^2^: correlation coefficient, RMSE: root mean square error. The larger R^2^ and the lower RMSE are, the better the prediction model is.

## Conclusions

This research revealed the close relationship between main volatile compounds of Chinese liquor and the response values obtained from electronic nose. Based on the response values, the identification of liquor ages was achieved by LDA with the total accuracy of 98.3% among 120 liquor samples, and PCA also performed well in liquor age discrimination. In addition, the possibility of using electronic nose combined with PLSR to predict liquor age and volatile compounds contents was finally confirmed. Our present findings provided a convenient alternative technique in quality assessment of Chinese liquor; however, more efforts should be made to work out an optimal combination of appropriate sensors and multivariate methods to promote the accuracy of the results.

## Materials and Methods

### Liquor samples and chemicals

Chinese liquor was provided by “Junchang” Liquor Factory, a very popular local brand located in Sichuan Province. Young liquor and liquors aged for 1, 2, 3, 4, and 5 years were used. All liquor samples were stored in sealed pottery at normal temperature before use. All reagents used for gas chromatography analysis were of analytical grade and bought from Donglilong Information Technology Co. Ltd., Lanzhou, China.

### Gas chromatography

Gas chromatography was performed using an Agilent 7890A gas chromatography (GC) unit equipped with a flame ionization detector (FID). All samples were analyzed on a LZP-950 column (50 m × 0.32 mm i.d., 1.0 μm film thickness), which is a specialty column for Chinese liquor gas chromatographic analysis. Pure reagents were firstly diluted with ethanol to give solution to ethanol ratio of 1:0, 1:1, 1:3, 1:7, 1:15, 1:31, 1:63 and 1:99, respectively, this series of diluted solution was used for the protraction of standard curves. Before the injection, all samples were filtered into a 2 mL autosampler vial using filtering membrance with the pore diameter of 0.45 μm to remove impurities. Then a measurement volume (1 μL) of the test sample was injected into GC from the autosampler vial with a split ratio of 1:1. The column carrier gas was nitrogen delivered at constant flow rate of 1 mL/min. The oven temperature was held at 65 °C for 8 min, then raised to 200 °C at a rate of 5 °C/min, and held at 200 °C for 50 min; injector and detector temperature were set at 230 °C and 250 °C respectively.

The identification analysis was made by comparing the retention times of volatile compounds in liquor samples with those corresponding reference compounds, and the quantification analysis was made using the established calibration curves. In order to eliminate the error caused by sample injection, amyl acetate was added into all samples as an internal standard substance, and the final quantification results were corrected using this internal standard. All liquor samples were analyzed and calculated in five replicates and the values were averaged.

### Electronic nose analysis

A PEN2 portable electronic nose (Airsense Analytics, Germany) was used in the experiment. The sensor array of this system was composed of 10 metal oxide semiconductors (MOS) type of sensors with different chemical compositions and thicknesses; each sensor generated specific response when exposed to corresponding volatile substances^25^. Namely, S1 (W1C, aromatic compounds), S2 (W5S, nitrogen oxides), S3 (W3C, ammonia), S4 (W6S, hydrogen), S5 (W5C, alkanes, less polar compounds), S6 (W1S, methane), S7 (W1W, sulphur compounds), S8 (W2S, alcohols), S9 (W2W, sulphur organic compounds) and S10 (W3S, high concentrations of methane). A data acquisition instrument was also equipped for collecting and recording response values.

3 mL of liquor sample was firstly diluted with deionized water to 300 mL, and then 5 mL of the diluted solution was transferred into a 500 mL beaker. After that, the beaker was sealed by preservative film and placed for 15 minutes to get the headspace reached equilibration. Detection time was set as 80 s, which was long enough for all sensors to reach stable response values. Afterwards, 100 s of cleaning (nitrogen) was executed to make sure the sensor array was normalized to the initial state. All liquor samples (20 replicates multiply by 6 groups) were detected under the same conditions at ambient temperature (25 °C).

### Statistical analysis

Relative standard deviation (RSD) of response values was calculated in order to assess their repeatability. One Way ANOVA was applied to select the most relevant sensors which were closely associated with liquor age. Only the high stable and relevant sensors were chosen as the database for the subsequent discrimination and prediction analysis. Since the original data of electronic nose was rather complicated, PCA was applied to reduce the dimensionality of the database, and scores plot was used for the grouping of liquor samples with different ages^26^. LDA has been proved to perform well in maximizing the variance between groups and minimizing the variance within groups^27^, therefore, it was employed in this research to investigate the accuracy of liquor age classification based on response values. PLSR is an effective regression method which incorporates the advantages of PCA, correlation analysis and multiple linear regressions^28^. In this research, PLSR was applied to predict liquor age and concentrations of volatile compounds using response values.

RSD, One Way ANOVA analysis, PCA and LDA were carried out using IBM SPSS Statistics 21.0. PLSR was performed using SAS 9.1.3.
